# Bridging Art and Algorithm: The Evolution of Plastic Surgery Across Eras

**DOI:** 10.1055/s-0046-1818625

**Published:** 2026-03-16

**Authors:** Ramesh Kumar Sharma

**Affiliations:** 1 Department of Plastic Surgery, Paras Hospital, Panchkula, Haryana, India; 2 Department of Plastic Surgery, Postgraduate Institute of Medical Education and Research, Chandigarh, India

**Keywords:** surgery, plastic reconstructive surgical procedures, cosmetic techniques, technological advancement, patient education, physician-patient relations

## Abstract

Over the past five decades, plastic surgery has transformed from a niche specialty into a widely accepted, technology-driven field. In the 1980s, the profession was defined by its emphasis on craftsmanship and authority, with a clear divide between the “respected realm” of reconstructive surgery and the “often-stigmatized” world of cosmetic procedures. The 1990s introduced minimally invasive techniques such as Botox and lasers, fostering a more collaborative surgeon–patient relationship. The 2000s saw the rise of digital imaging and the internet, empowering patients and shifting decision-making dynamics. In the 2010s, the integration of prevention, wellness, and mental health became central to practice. By the 2020s, advancements like bioprinting and AI-enabled personalized treatments emerged, raising new ethical considerations. Today, plastic surgery is democratized and mainstream, combining surgical expertise, regenerative medicine, and psychological support. Surgeons now serve as partners in patients' identity journeys, balancing empowerment with ethical responsibility.

## Introduction

Plastic surgery has evolved significantly over the past 45 years, transitioning from an exclusive specialty to a widely accepted, technology-driven discipline. In the early 1980s, reconstructive procedures were predominant, while cosmetic surgery remained a discreet practice. Today, cosmetic interventions are mainstream and increasingly regarded as essential, influenced by social media trends. As patients have become better informed, surgeons play a critical role in setting realistic expectations and guiding decision-making. The changing trends, both societal and technological, as discussed below, would help us understand the sea change that this specialty has undergone in the last few decades.

## The 1980s: Foundations in Craft and Authority

When I began training in plastic surgery in the early 1980s, the field was exclusive and closeted. Procedures involved large incisions, basic tools, and often required multiple stages with long recovery times. Every operation was carefully planned and executed with precise tissue handling, guided by the principle of balancing blood supply and aesthetics. Training was rooted in true mentorship, where students learned both the art and science of surgery from their teachers with deep respect and dedication. Reconstructive surgery was respected as noble work. The surgeon was seen as an artist and an authority, and trust in the surgeon was absolute. Cosmetic surgery was a status symbol for the wealthy but often viewed with suspicion or disdain by others. Surgery for burn victims, cleft palates, or post-mastectomy reconstruction was seen as noble and legitimate medicine.

In those early days, people learned about plastic surgery mainly through discreet referrals or private meetings with a single trusted surgeon—there was no idea of comparing different options. Patients put complete faith in their surgeon's skills. With passing time, the field was quickly advancing. Old methods like skin tubes and grafts were being replaced by newer techniques using skin and muscle flaps, and a better understanding of blood supply made more complex tissue transfers possible. With the introduction of finer instruments and the operating microscope, surgeons began performing free microvascular tissue transfers. By 1990, both re-implantations and free tissue transfers had become standard in teaching hospitals across the country.

## The 1990s: Minimally Invasive Innovations

In the 1990s, plastic surgery scenario changed with the arrival of Botox, fillers, and lasers. These new treatments enabled people improve their appearance without major surgery or long recovery times. After initial skepticism, these procedures quickly gained popularity and became essential for both cosmetic and reconstructive plastic surgery. This era also saw a change in the relationship between patients and surgeons: instead of simply following the surgeon's recommendations, patients became more involved in discussing their goals and preferences.

## The 2000s: Digital Transformation

With the arrival of digital imaging, patients could now preview potential results before surgery, which although helped manage expectations, but also introduced new challenges. The internet gave patients unprecedented access to information—both accurate and misleading—empowering them to make more informed choices but also reducing the traditional authority of the surgeon. This era marked a shift toward greater transparency and patient autonomy in the decision-making process.

## The 2010s: Prevention and Wellness

Before the 2010s, plastic surgeons mainly focused on correcting specific issues, such as facelifts, breast augmentation, or rhinoplasty. In the 2010s, the field shifted to promoting overall wellness and prevention. Surgeons began offering preemptive treatments to younger patients and incorporated elements of dermatology, nutrition, and mental health into their practices. Surgery became just one of several tools to help patients achieve their goals. This holistic approach redefined the surgeon's role, emphasizing long-term well-being and making them partners in their patients' journeys toward health and self-confidence.

## The 2020s: Personalization and Ethical Frontiers

In recent years, breakthroughs such as bioprinting and AI-assisted planning have made it possible to deliver highly personalized treatments in plastic surgery. However, these innovations have also introduced complex ethical dilemmas. For example, algorithms can now recommend what they consider “optimal” facial features based on cultural data, which has shifted the surgeon's role from being solely a technical expert to acting as a mediator between technology and human values. As a result, consultations today often extend beyond medical considerations to include thoughtful discussions about identity, ethics, and the true meaning of beauty. The central question has evolved from simply asking, “Can we do this?” to a deeper reflection: “Should we do this?”

## 2026 and Beyond: Integration, Identity, and Democratization

Today, plastic surgery is widely accepted as a form of self-improvement. Social media has helped reduce stigma but has also contributed to uniform beauty standards and body image concerns. The field now blends surgery, regenerative medicine, and mental health support, with surgeons guiding patients to balance their inner identity with their outward appearance. The impact of these procedures depends on ethical practice and informed consent, making the surgeon's guidance more important than ever.

## Transitioning for a 1980s surgeon: Adapting for the Future

For surgeons who began their careers in the 1980s, staying relevant today means embracing new technologies, collaborating with younger colleagues, and shifting from being the sole authority to a more collaborative role. Patient consultations now often address the influence of social media and digital filters on self-image. While technology is a powerful tool, it should enhance—not replace—surgical skill and judgment. Experience and wisdom from earlier eras remain valuable assets, serving as a bridge between traditional artistry and modern innovation.

## Messages for the Next Generation

True artistry in plastic surgery is measured over decades, not fleeting trends. Empowering patients must always be balanced with ethical responsibility, and technology should be used to enhance individuality, not erase it. Younger surgeons must master both digital tools and anatomical expertise, act as empathetic guides, and uphold ethical standards in an increasingly commercialized field.

## Conclusion

The evolution of plastic surgery reflects broader societal changes. The future will belong to those who can blend the enduring values of the past with innovative new techniques—enhancing not just appearances, but lives. For surgeons trained in the 1980s, today's world of plastic surgery may seem almost unrecognizable, though core skills like anatomy and patient care remain vital. Adapting to the present means building on your experience rather than discarding it. Modern surgeons are shifting from being the sole authority to becoming collaborative guides, helping patients interpret research and make informed choices. Continuous learning is essential—embrace digital technologies, seek targeted training, and be open to learning from younger colleagues. Technology should enhance, not replace, surgical craftsmanship. The wisdom gained from earlier eras remains an asset in today's rapidly changing field.

